# Cutaneous Neonatal Lupus Erythematosus: A Case Report

**DOI:** 10.7759/cureus.2212

**Published:** 2018-02-21

**Authors:** Kamleshun Ramphul, Stephanie G Mejias, Yogeshwaree Ramphul-Sicharam

**Affiliations:** 1 Department of Pediatrics, Shanghai Xin Hua Hospital Affiliated to Shanghai Jiao Tong University School of Medicine, Shanghai, People's Republic of China; 2 Department of Pediatrics, Robert Reid Cabral Children's Hospital Affiliated to the University Iberoamericana Unibe School of Medicine; 3 Sir Seewoosagur Ramgoolam National Hospital

**Keywords:** neonatal lupus

## Abstract

Cutaneous neonatal lupus erythematosus (NLE) is a rare condition caused by the passive transfer of autoantibodies from mother to fetus. The disease most commonly presents itself with multiple erythematosus annular lesions or arcuate macules. In 10% of the cases, a cardiac anomaly can coexist. Appropriate diagnosis and laboratory and cardiac testing should be done to rule out any complications. Treatment usually includes topical steroids, following which the lesions will disappear after about eight weeks with no sequelae. This research work deals with a case of cutaneous NLE in a two-week-old male.

## Introduction

Cutaneous neonatal lupus erythematosus (NLE) is a rare condition that was first described in 1954 by Dr. McCuiston [1]. It is an autoimmune condition caused by the passive transfer of autoantibodies from mother to fetus. The main features of the cutaneous form usually include, most commonly, multiple erythematosus annular lesions or arcuate macules. The rash is often described as a raccoon-eye appearance. The face is the most common site affected, although the palms, soles, or diaper area can be concerned as well [2-3]. Differential diagnosis and appropriate treatment should be started to prevent further complications. Other complications related to NLE such as cardiovascular block and renal functions should be ruled out.

We present a case report of a newborn with cutaneous neonatal lupus erythematosus at our hospital.

## Case presentation

**Figure 1:**
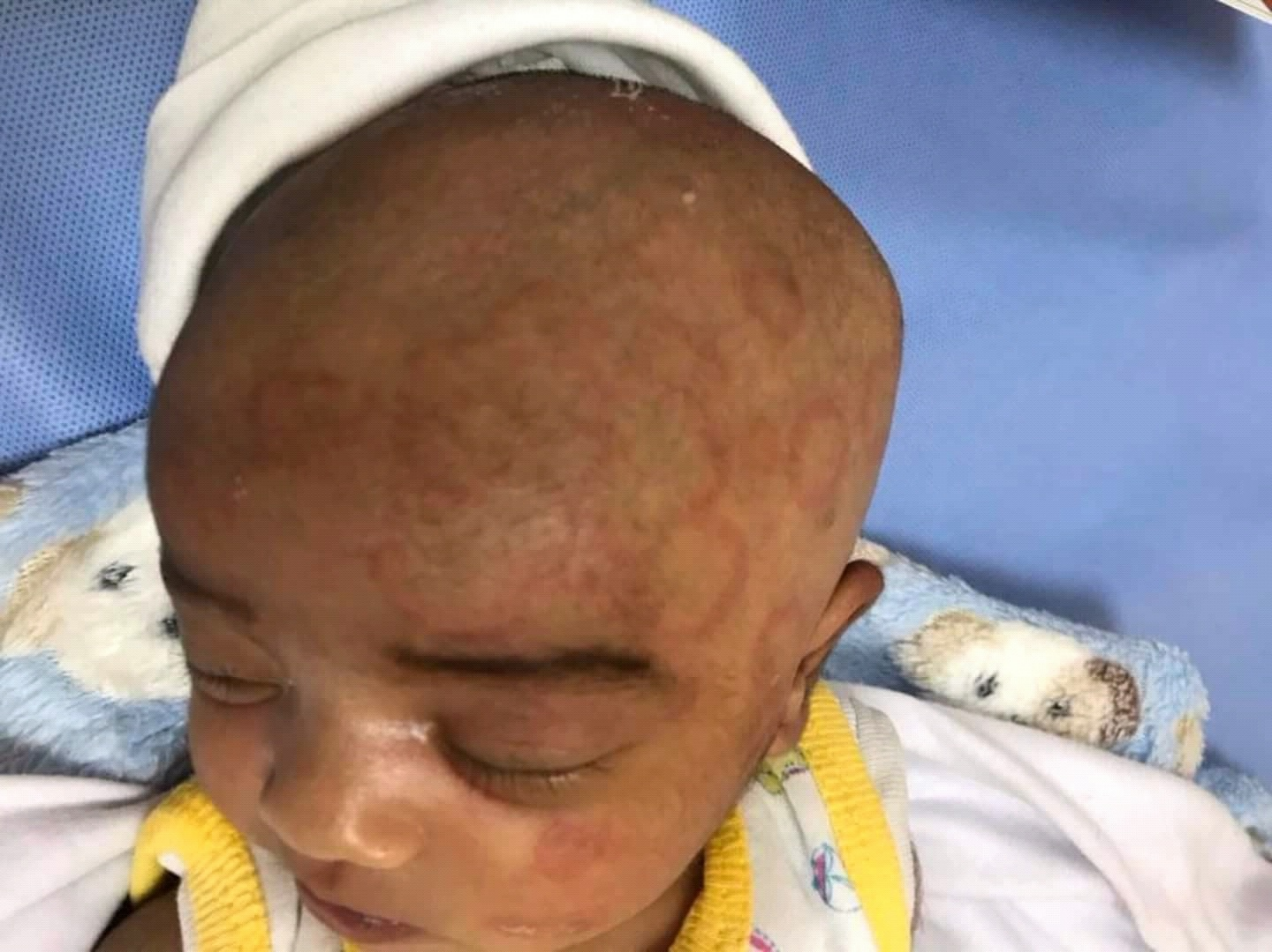
Two-week-old male with multiple cutaneous erythematosus annular lesions over the face and scalp

A two-week-old, Iraqi, male newborn was brought to the pediatric unit for a new onset of multiple cutaneous erythematosus annular lesions over the face and scalp for the past one week, as seen in Figure 1. The lesions were progressively increasing in size. There was no history of fever and the baby was feeding well. No vaccination had been done yet and there was no exposure to any sick contacts or animals.

The mother had one spontaneous abortion in the past. The recent pregnancy was uneventful, and the baby was born from a spontaneous vaginal delivery at week 36. The birth weight was 2.6 kg and the Apgar score was nine. The mother was previously tested positive for systemic lupus erythematosus (SLE) three years ago. She was on low-dose systemic corticosteroids and hydroxychloroquine. She denied any flares during pregnancy.

On physical examination of the patient, no other systemic manifestations were found. The lungs were cleared and the abdomen soft and nontender with no hepatosplenomegaly. Moreover, there was no evidence of any viral or bacterial illness.

The laboratory findings showed normal white blood cell counts and no evidence of inflammation was found. The diagnosis of cutaneous NLE was made, and the mother of the baby requested for further laboratory examinations. The mother was tested positive for antinuclear antibodies (ANA) 1:2560, anti-Sjögren’s-syndrome-related antigen A (Anti-SSA/Ro), anti-Sjögren’s-syndrome-related antigen B (anti-SSB/La), and anti-U1 ribonucleoprotein (anti-U1 RNP). The baby’s serum was tested positive for ANA of more than one in 1380 and the anti-SSA/Ro and anti-SSb/La antibodies. Liver enzymes and renal function tests in both mother and baby were normal. An echocardiogram showed no cardiac anomalies or cardiomyopathy in the baby.

Treatment with mild topical steroids was prescribed and the mother was advised to avoid sun exposure. We also reassured the mother that these lesions would disappear once the maternal antibodies were removed over time from the neonatal circulation.

The child presented to the clinic for two follow-ups; after one month and after three months. We noticed a gradual improvement in the rashes and at three months, all of his lesions had cleared. Liver and kidney function tests were all normal and the complete blood cell count showed no signs of inflammation.

## Discussion

NLE is a rare autoimmune condition found in the newborns of mothers with a history of autoimmune conditions and having autoantibodies against Ro, La, and U1-Ribonucleoprotein. The main manifestation is a cutaneous lesion [4] and there is a risk of cardiac complications [5]. Only 10% of cases with both cardiac and skin lesions have been reported in the past. Other possible complications are hepatic, renal, and hematological abnormalities, and proper laboratory tests should be done to rule them out. The risk of heart block is believed to be from the binding of the anti-Ro/SSA and Anti-La/SSB autoantibodies to the neonate’s cardiac cells, leading to possible remodeling and fibrosis [6]. Damage to the L and T type calcium channels is also possible, and this can affect the atrioventricular (AV) and sinoatrial (SA ) nodes [7].

A prenatal evaluation of maternal anti-Ro/SSA and Anti-La/SSB antibodies in patients with SLE or other autoimmune conditions, such as Sjögren syndrome and rheumatoid arthritis should be done in the early stages of pregnancy and then repeated at the end of the first trimester. Fetal monitory for any possible heart block should also be performed in mothers with high levels of antibodies and some experts advice performing a weekly pulsed-Doppler fetal echocardiography for such patients from week 18 to 26 [8].

The differential diagnosis should include other similar skin conditions, such as urticaria, tinea corporis, seborrheic dermatitis, and the annular erythemas of childhood among many others. The treatment of skin lesions usually includes topical steroids, and they resolve by eight months with any sequelae.

## Conclusions

Cutaneous NLE is a rare condition resulting from the passive transfer of autoantibodies from the mother during fetal life. The present patient did not have any coexisting cardiac or systemic abnormalities. A gradual improvement in the rashes over three months was noted with topical steroid therapy. Although the prognosis of cutaneous NLE is usually excellent, proper measures should be taken to rule out conditions with similar cutaneous manifestations and the presence of any coexisting systemic disease.

## References

[1] McCuistion CH, Schoch EP Jr (1983). Possible discoid lupus erythematosus in newborn infant. Report of a case with subsequent development of acute systemic lupus erythematosus in mother. Arch Derm.

[2] Lee LA (2009). The clinical spectrum of neonatal lupus. Arch Dermatol Res.

[3] Cimaz R, Spence DL, Hornberger L, Silverman ED (2003). Incidence and spectrum of neonatal lupus erythematosus: a prospective study of infants born to mothers with anti-Ro autoantibodies. J Pediatr.

[4] Shiiya C, Ota M (2017). Facial rash, fever, and anemia in a newborn. JAMA.

[5] Sommer LL, Manders SM (2015). Seeing double: annular diaper rash in twins. Pediatr Dermatol.

[6] Clancy RM, Neufing PJ, Zheng P, O'Mahony M, Nimmerjahn F, Gordon TP, Buyon JP (2006). Impaired clearance of apoptotic cardiocytes is linked to anti-SSA/Ro and -SSB/La antibodies in the pathogenesis of congenital heart block. J Clin Invest.

[7] Garcia S, Nascimento JH, Bonfa E, Levy R, Oliveira SF, Tavares AV, de Carvalho AC (1994). Cellular mechanism of the conduction abnormalities induced by serum from anti-Ro/SSA-positive patients in rabbit hearts. J Clin Invest.

[8] Donofrio MT, Moon-Grady AJ, Hornberger LK (2014). Diagnosis and treatment of fetal cardiac disease: a scientific statement from the American Heart Association. Circulation.

